# Deciphering the Blood-Brain Barrier Damage in Stroke: Mitochondrial Mechanism

**DOI:** 10.4172/2314-7326.S2-e002

**Published:** 2015-08-22

**Authors:** Xuefang Ren, James W. Simpkins

**Affiliations:** Department of Physiology and Pharmacology, Experimental Stroke Core, Center for Basic and Translational Stroke Research, West Virginia University, Morgantown, West Virginia, USA

## Introduction

Stroke is a complex vascular and neurological syndrome that can lead to death and disability [1]. About 15 million people worldwide [1] and 800,000 people in the United States suffer stroke each year [2]. On average, in the U.S. every 40 seconds someone has a stroke and every 4 minutes someone dies from a stroke [2]. This medical emergency has very limited treatments, and causes serious health and economic burdens in the United States and globally.

Blood-brain barrier (BBB) is composed of highly specialized cerebrovascular endothelial cells, seals brain tissue from the circulating blood, and prevents blood, bacteria or toxins from reaching the central nervous system (CNS). In acute ischemic stroke, BBB is disrupted, and blood solutes penetrate into the CNS parenchymal extracellular space then cause cerebral edema [3]. Although much has been observed in this stroke-induced brain edema, such as inflammatory infiltration, releasing of chemokines and cytokines, much less has the mechanism of BBB disruption been understood.

Enrichment of mitochondria in the BBB capillaries has been observed 3 decades ago [4]. It has been recognized that mitochondria involve in maintaining of ion differentials and transportation of chemicals between blood and brain extracellular fluid. We have recently demonstrated that mitochondria are crucial for shaping the cerebral endothelial capillary architecture and the integrity of the BBB [5]. Mitochondrial blockade rapidly opens the BBB both in vitro cell culture model and in vivo epidural application model, increases BBB permeability, and exacerbates stroke outcomes in acute experimental stroke in mice [5].

It is estimated that 30% to 40% stroke patients had recent acute infections [6]. Post-stroke infections worsen outcomes in patients [6,7]. Infection mimic, bacteria-derived lipopolysaccharide exacerbates stroke outcomes in experimental stroke models [5,8,9]. McColl et al. [9] reported that lipopolysaccharide challenge induces inflammatory responses, activates immune cells to release of inflammatory cytokines and chemokines, which further act on vascular endothelial system and exacerbate brain damage in murine experimental stroke. However, our work reveals that lipopolysaccharide directly acts on its receptor, toll-like receptor 4 (TLR4), communicates with TLR4 signal pathways, and compromises mitochondrial oxidative phosphorylation on BBB endothelium [5]. Mitochondrial damage has also been documented in ischemic stroke patients [10,11] and experimental stroke models [12,13]. The “double attack” from stroke and infection drives endothelial mitochondria in a direction unfavorable to the functional BBB and worsens stroke outcomes.

Mitochondria participate in generation of ATP and maintenance of body temperature [14]. Is body temperature affected if the endothelial mitochondria are compromised by infections/stroke? The answer is yes. Usually, you might feel cold at first during an infection, and this hypothermia is followed by hyperthermia. Therefore, the first response to infections/stroke could be a direct mitochondrial impairment that may cause hypothermia, then the second is the induced cytokine productions (some of them are known “pyrogens”), which are well studied in infections- or stroke-induced inflammatory responses and lead to hyperthermia.

It is expected that targeting of BBB endothelial mitochondria protects BBB damage in stroke. Estrogen increases cerebrovascular expression of several mitochondrial respiratory chain proteins, including cytochrome c and complex IV subunits [15]. Estrogen has been shown to reduce BBB permeability [16] and attenuate stroke damage [17]. Mitochondrial failure is observed in the form of reduced activity of respiratory chain complex I, III and IV during post cerebral ischemia/reperfusion injury [18]. Other mitochondrial protecting drugs specifically targeting these complexes in BBB endothelium may alleviate BBB damage and improve stroke outcomes as well.

Treatments for ischemic stroke are currently limited to an FDA-approved drug, recombinant tissue plasminogen activator (tPA) within 4.5 hour therapeutic window [19]. However, tPA does not target endothelial mitochondria or inflammatory responses. Unveiling of this mitochondrial mechanism may lead to the successful development of novel therapeutic strategies aimed at endothelial mitochondria. This approach will be beneficial for the large populations of stroke patients, and other BBB involved neurological diseases.
